# sFLT01 modulates invasion and metastasis in prostate cancer DU145 cells by inhibition of VEGF/GRP78/MMP2&9 axis

**DOI:** 10.1186/s12860-021-00367-5

**Published:** 2021-05-19

**Authors:** Sepideh Taghizadeh, Zahra-Soheila Soheili, Mehdi Sadeghi, Shahram Samiei, Ehsan Ranaei Pirmardan, Ali Kashanian, Fahimeh Zakeri, Hamid Latifi-Navid, Hoda Shams Najafabadi

**Affiliations:** 1 Department of Molecular Medicine, National Institute of Genetic Engineering and Biotechnology, 14965/161, Tehran, Iran; 2 Blood Transfusion Research Center, High Institute for Research and Education in Transfusion Medicine, Tehran, Iran; 3 Molecular Biomarkers Nano-Imaging Laboratory, Brigham and Women’s Hospital, Department of Radiology Harvard Medical School, Boston, MA USA

**Keywords:** Prostate cancer, Angiogenesis, sFLT01, VEGF, GRP78

## Abstract

**Background:**

About 90% of cancer-related deaths are due to metastasis of cancer cells, and angiogenesis is a critical step in this process. sFLT01 is a novel fusion protein and a dual-targeting agent that neutralizes both VEGF and PlGF proangiogenic activities. GRP78 dual effect in tumor growth and angiogenesis could be activated under VEGF stimulation. The current study was designed to investigate the inhibitory impact of sFLT01 protein on VEGF/GRP78 axis. To this point, sFLT01 construct was synthesized, recombinant plasmid was expressed in eukaryotic host cells, sFLT01-HisTag protein was extracted and analyzed. The functional activity of sFLT01 on VEGF-enhanced tube formation and angiogenesis of HUVEC cells were examined. Eventually, the inhibitory impact of sFLT01 on growth, invasiveness, and migration of human prostate cancer cell line, DU145, was assessed. Real-time PCR evaluated the level of GRP78 and its effect on the downstream factors; matrix metallopeptidase proteins 2&9 (MMP2&9) along with tissue inhibitor of metalloproteinase proteins1&2 (TIMP1&2) under sFLT01 stimulation.

**Results:**

According to the data, sFLT01 protein showed modulatory impact on proliferation, invasion, and migration of DU145 cells along with the potential of HUVECs angiogenesis. Real-Time PCR analysis depicted a significant downregulation in GRP78, MMP2 and MMP9 transcripts’ levels, and a subsequent elevation of TIMP1 and TIMP2 expression under sFLT01 stimulation was detected.

**Conclusion:**

Overall, these data indicated that the inhibitory impact of sFLT01 on cancer cells growth and invasiveness could be mediated through the modulation of VEGF/GRP78/MMP2&9 axis and activation of TIMPs.

## Background

Prostate cancer (PC) is a malignant growth of prostate cells with little symptoms and ranked as the second deadliest cancer worldwide. According to the cancer statistics, over 6000 new PC cases have been diagnosed each year in the united states of America [1]. Although the PC population in Asian countries is still low, the incidence rate of PC has been reported to increase faster than the USA and European countries due to the lifestyle alteration [2].

About 90% of cancer-related deaths are due to metastasis of cancer cells from the primary tumor to other organs [3]. PC is the most common non-skin malignancy among men, and since the advent of PSA testing, most patients have been diagnosed with topical PC and chemotherapy-treated with Docetaxel and Prednisone drugs [4, 5]. However, not all of the patients are fully recovered. Hence, ongoing efforts are being made to design new therapeutic strategies for targeting the PC.

Angiogenesis is a critical step in cancer metastasis and could be stimulated by various factors, including the vascular endothelial growth factor (VEGF). During the angiogenesis, extracellular membrane of the body vessels is degraded quickly, and new capillaries emerge by the enhanced level of highly activated endothelial cells. Up today, multiple intra and extracellular signaling factors have been introduced that are playing important roles in tumor angiogenesis.

Among them, VEGF is reported to be directly associated with cancer cells proliferation, metastasis, angiogenesis, and chemotherapy resistance in various human cancers [6]. Anti-VEGF agents that neutralize VEGF in some animal model describe inhibitory effects on proliferation and metastatic dissemination in solid tumors [7]. In cancerous tumors, GRP78 expression could be provoked due to the hypoxia increased glucose demand or glycolytic activity and this, supplies the cells with glucose and oxygen [8]. This heat-shock protein is located in the endoplasmic reticulum (ER) lumen and regulates protein folding and transportation along with ER homeostasis and responses to stress signals such as accumulation of unfolded proteins [9, 10].

Glucose-regulated protein 78 (GRP78) is reported to be associated with angiogenesis and metastasis of cancer tumors directly. Knockdown of GRP78 suppresses VEGF- signaling and endothelial cell proliferation. Yet, VEGF induces expression of cell surface GRP78 in endothelial cells [11]. MMPs or matrix metalloproteinases are a group of zinc-binding proteins that involve in the degradation of ECM components, the tumor surface and the basement membrane, which causes tumor cell migration into other tissue. MMPs along with EMT play key role to promote angiogenesis and metastasis [12]. The positive impact of GRP78 on MMP2 expression has been previously described in hepatocyte carcinoma cells, in which GRP78 knocking down, reduced the MMP2 level and activity via ERK/JNK signaling pathway suppression [13]. Also, it has been shown that the ERK signaling pathway stimulates MMP2 and MMP9 activity though triggering the ADAM17, a metalloprotease enzyme highly expressed in various human disorders, including cancers [14].

Accordingly, most of the chemotherapies in patients with advanced cancer are based on using angiogenesis inhibitors such as Bevacizumab, Thalidomide, and its analogs, kinase inhibitors like Sorafenib and Cediranib (AZD2171), and other angiogenic signaling repressors [15].

It has been reported that PC cells widely express VEGF protein, and the sera level of VEGF in patients with metastatic PC is higher than in patients with early PC [16]. Therefore, neutralizing VEGF may be considered as a promising strategy for angiogenesis inhibition. One of the VEGF blockers is sFLT01; a secreted chimeric protein consists of an FMS-like tyrosine kinase (FLT) domain of VEGFR1, the Glycine linker, and an Fc domain of IgG1 [17]. sFLT01 acts as a soluble receptor and inhibits angiogenesis by blocking the VEGF protein along with increasing the endothelial cell attachment to the extracellular matrix. Moreover, this protein may also have acceptable safety profiles [18]. The strong antitumor effect of sFLT01 was demonstrated in several xenograft models. Intraperitoneal injection of vehicle or sFLT01 (10 or 25 mg/kg) was performed twice per week in mice bearing SC H460 NSCLC, HT29 colon carcinoma, Karpas 299 lymphoma, or MOLM-13 AML tumors [17]. The current study was designed to investigate the anticancer effects of sFLT01 protein on the proliferation and invasiveness of the PC cell line.

### Statistical analysis

All statistical analyses were performed with IBM SPSS Statistics software version 22 (IBM, USA). Shapiro-Wilk normality test was used for data normalization. Significant statistical differences were calculated by one/two-way analyses of variance (ANOVA) and Tukey’s multiple comparison tests. The data were depicted as the mean ± SD (Standard deviation) and considered as significant if *P* < 0.05 (*).

## Results

### Cloning, transfection, purification, and analysis of recombinant sFLT01 protein

**Fig. 1:**
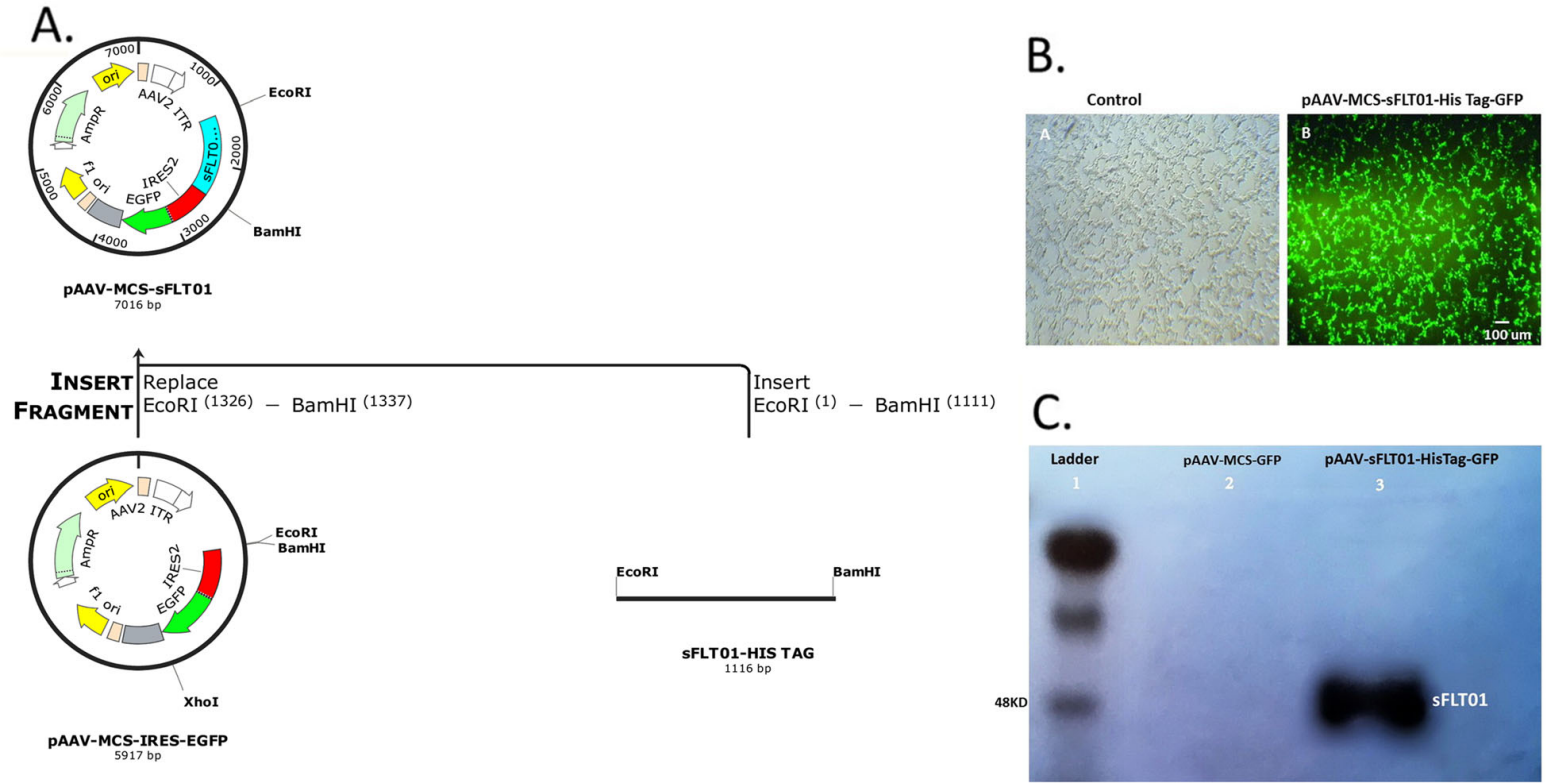
Cloning of the sFLT01, vector transfection and western blot analysis. **a** The polyhistidine-tag was added at the end of sFLT01 sequence, and cloned into pAAV-MCS-GFP plasmid. SFLT01-HisTag fragment digestion examined with the EcoRI and BamHI enzymes, resulted in 1116 bp fragment (sFLT01-HisTag) indicating that the target sequence was inserted in the vector body. **b** Transfection of DU145 cells with the recombinant pAAV-sFLT01-HisTag-GFP plasmid showed more than 90% of the cells were positive for GFP, (**a**): DU145 cells; no transfection, (**b**): GFP over expression in DU145 cells which had been transfected by pAAV-sFLT01-His Tag-GFP. **c** CM of HEK293T cells that had been transfected by pAAV-sFLT01-HisTag-GFP was collected 72 h post transfection, purified with nickel affinity chromatography column and applied in western blot experiment by the human VEGFR1/Flt-1 primary antibody

The final sFLT01-HisTag sequence (1116 bp) was inserted into a pAAV-MCS-GFP plasmid (5900 bp), and resulted in a pAAV-sFLT01-HisTag-GFP plasmid, 7 kb length (Fig. 1a). The recombinant plasmids were then amplified and examined with the EcoRI/BamHI enzymes.

After verification of gene cloning, the pAAV-sFLT01-HisTag-GFP plasmid was transfected into the DU145 cells for sFLT01-HisTag production and analysis. DU145 cultures that had been treated with media and DU145 cultures that had been transfected by pAAV-MCS-GFP recruited as controls for the pAAV-MCS-sFLT01transfected cultures. 48 h after transfection, cells were examined for GFP expression and revealed that cells were positive for GFP (Fig. 1b).

To extract the sFLT01 protein for further analysis, CM from HEK293T cells, transfected with pAAV-sFLT01-HisTag-GFP, was collected 72 h after transfection, purified with nickel affinity chromatography and determined using western blotting (Fig. 1c).

### Impact of sFLT01 protein on prostate cancer cells viability

**Fig. 2:**
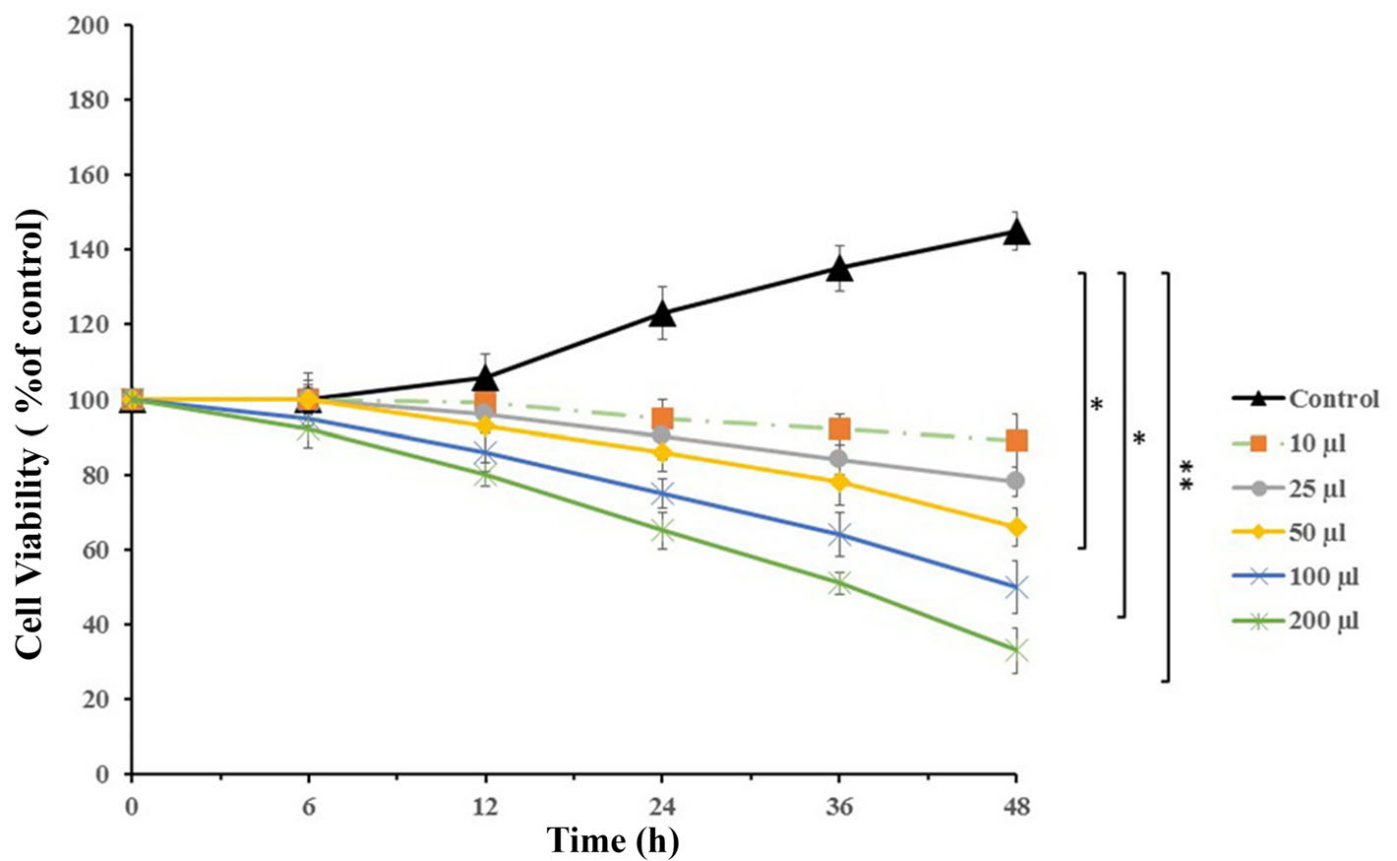
Cell viability analysis. Prostate cancer DU145 cells were incubated with various doses of conditioned media containing sFLT01 protein for 48 h. Cell density was analyzed using the MTT assay method and measured by a microplate reader at 580 nm wavelength. Cellular viability of the treated cells was 50% lower than the control group (*P* < 0.05)

Cellular viability was determined by analyzing the activity of mitochondrial succinate dehydrogenase enzyme. The target groups consisted of DU145 cells treated with various doses of CM collected from the DU145 cells (10–200 μl/well), and a control group of cancer cells incubated with RPMI1640 media. According to the data, cellular viability of the cancer cells treated with 100 μl of CM was about 50% lower than the control group (*P* < 0.05, Fig. 2). This dose was chosen for future examinations.

### sFLT01 treatment reduced tube formation ability in HUVECs

**Fig. 3:**
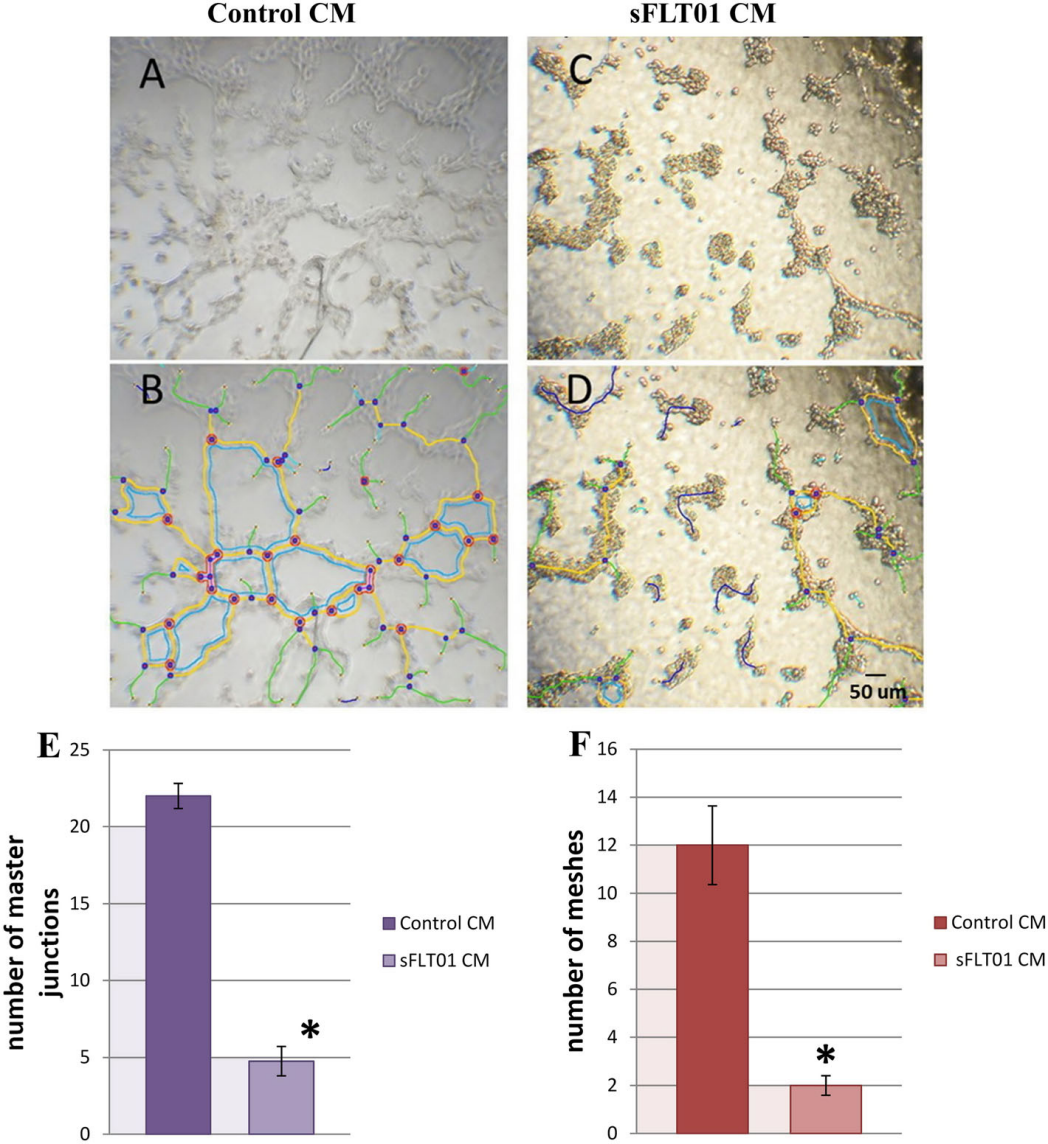
Analysis of sFLT01 effect on tube formation ability in HUVECs. Tube formation assay was performed on the human umbilical vein endothelial cells (HUVECs)**.** Cells were seeded into a 96-well plate, pre-coated with Matrigel, and treated with conditioned media containing sFLT01 CM and control CM for 18 h. **a** Representative phase contrast picture of tubular network of HUVEC cells in control group. **b** Corresponding skeletons of tubular networks identifying segments (yellow color), mesh (blue color) and nodes (red color) by AngioTool plugin (ImageJ software) in control group. **c** Representative phase contrast picture of tubular network of HUVEC cells in sFLT01 conditioned media group. **d** Corresponding skeletons of tubular networks in sFLT01 group. **e** Quantification of the number of master junctions and (**f**) meshes using imageJ software. Four randomly picked field from different wells were evaluated for the number of junctions and closed meshes (*n* = 4*P < 0.05)

Considering this point that VEGF is a known angiogenesis factor and Matrigel develops an angiogenic natural response, the suppressive effect of sFLT01 protein on formation of capillary structures in HUVEC cells was examined (Fig. 3). According to the data, sFLT01 treatment significantly suppressed VEGF-enhanced tube formation, in a way that the number of master junctions in the sFLT01 CM group was 4.63 fold lower than control (treated by CM from pAAV-MCS-GFP transfected cells, *P* < 0.05). Similar result was obtained from analyzing the number of meshes between target and control groups; in which sFLT01 containing CM reduced meshes population about 6 fold in sFLT01 treated HUVECs versus control HUVEC cells (*P* < 0.05).

### Impact of sFLT01 protein on invasiveness and migration of prostate cancer cells

**Fig. 4:**
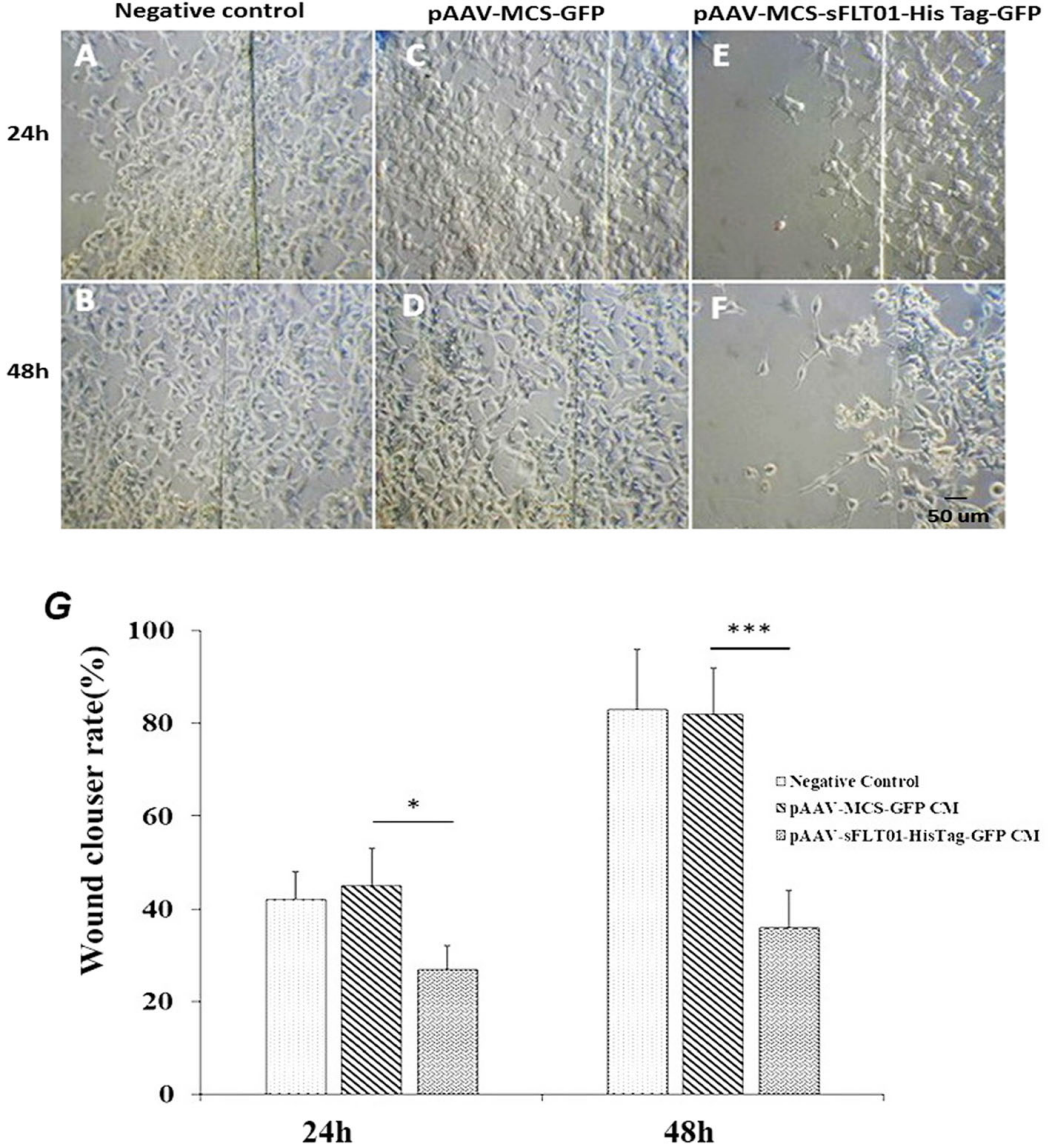
Wound healing assay. Cells were treated with 100 μl of conditioned media from DU145 cultures that had been transfected by sFLT01containing construct and/or control consteucts. After 24–48 h incubation in humidified CO2 incubator, cultures were fixed in 3% formaldehyde solution and visualized by a light microscope. **a**, **b** DU145 cells migration as a negative control. **c**, **d** migration of DU145 cells which had been treated by CM from pAAV-MCS-GFP / control cultures. **e**, **f** migration in DU145 cultures which had been treated by CM from pAAV-sFLT01-HisTag-GFP transfected cultures. **g** data indicates that following treatment with sFLT01 protein containing CM for 24 h and 48 h, the mobility of DU145 cells was significantly decreased versus the control group (treated by CM from pAAV-MCS-GFP transfected cells); 0.43-fold (*P* < 0.05) versus 2.27-fold (*P* < 0.001)

**Fig. 5:**
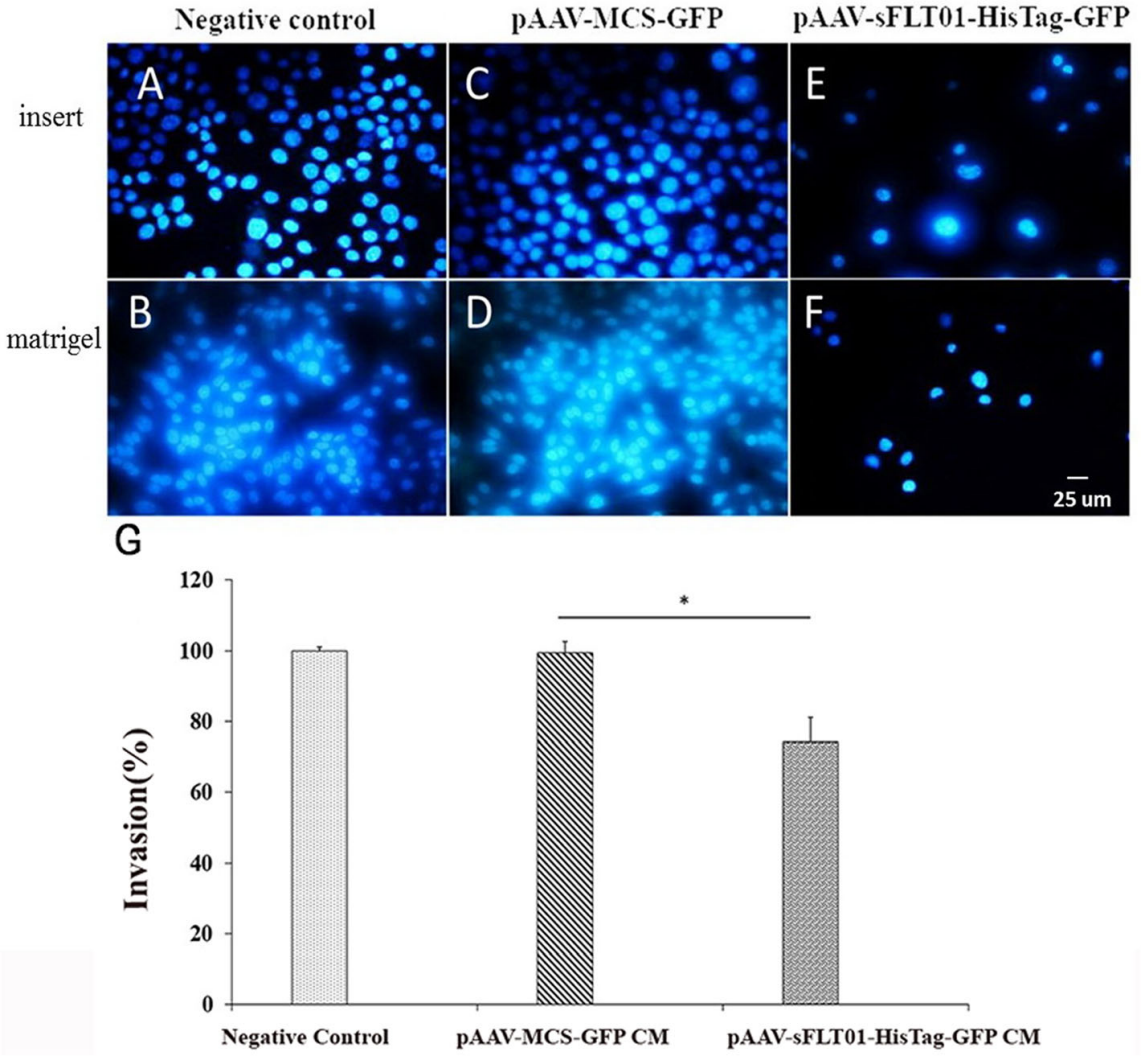
Cell invasion assay. DU145 cells were seeded in matrigel-coated transwell chambers. 5 × 10^5^ DU145 cells were transfected by pAAV-sFLT01-HisTag-GFP and/or control (pAAV-MCS-GFP transfected DU145 cells). Serum-free media was prepared and added to the matrigel-coated transwell chambers. The chambers were then placed in the lower plate which had been filled with 10% FBS containing media, After 24 h the non-invading cells were removed, membranes were fixed in methanol, and stained with DAPI. **a** Dapi staning of DU145 cells on insert (without matrigel), (**B**) Dapi staning of DU145 cells on insert coated with matrigel. **c** Dapi staning of DU145 cells which had been transfected by pAAV-MCS-GFP, as a control. **d** Dapi staning of DU145 cells which had been transfected by pAAV-MCS-GFP, on insert coated by matrigel as control. **e** Dapi staning of DU145 cells which had been transfected by pAAV-sFLT01-HisTag-GFP, on insert. **f** Dapi staning of DU145 cells which had been transfected by pAAV-sFLT01-HisTag-GFP, on insert coated matrigel. Data showed that sFLT01 inhibited invasiveness of DU145 cells compared to control. **g** Each value is the mean ± SD of three independent experiments. The asterisks indicate significantly different from the control group

Wound healing assay and cell invasion analysis were performed to estimate the impact of sFLT01 protein on the migration and invasiveness of PC cells. The data indicated that following the treatment with CM media containing sFLT01 protein for 24 h and 48 h, the mobility of DU145 cells was significantly decreased versus the control group (treated by CM from pAAV-MCS-GFP CM transfected cells); 0.43-fold (*P* < 0.05) and 2.27-fold (*P* < 0.001), respectively (Fig. 4). Accordingly, the invasion of the cancer cells incubated by pAAV-sFLT01-HisTag-GFP transfected cells was statistically lower than the control group (1.34-fold, *P* < 0.05, Fig. 5). These observations showed that the sFLT01 protein imposed a negative impact on the invasiveness and migration of DU145 cells.

### Modulatory effect of sFLT01 protein on the expression of VEGF signaling mediators

**Fig. 6:**
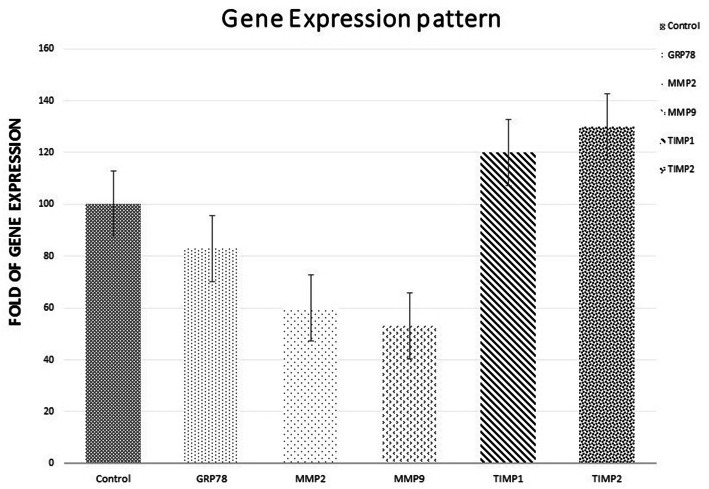
Expression of selected genes. mRNA levels of the interested genes were measured and adjusted to GAPDH 36 h after transfection. Data showed that in sFLT01 treated DU145 cultures GRP78, MMP2, and MMP9 decreased about 17, 40, and 43%, respectively. However, TIMP1 (20%) and TIMP2 (30%) revealed increased expression in treated cultures

To investigate the impact of sFLT01 protein on VEGF expression in DU145 cells, the level of glucose-regulated protein 78 (GRP78), matrix metallopeptidase protein 2 & 9 (MMP2&9), and tissue inhibitor of metalloproteinase protein 1 & 2 (TIMP1&2) were evaluated (Fig. 6). Real-time PCR data in DU145 cells transfected by pAAV-sFLT01-HisTag-GFP showed that expression of GRP78, MMP2, and MMP9 were downregulated about 17, 40, and 43%, respectively when compared to control cultures that had been transfected by pAAV-MCS-GFP. However, upregulation was detected for TIMP1 (20%) and TIMP2 (30%), under sFLT01 stimulation.

## Discussion

Tumor formation is a multi-step process including survival, proliferation, angiogenesis, migration, and invasion. It is regulated by multiple cell signaling pathways [19]. Since, angiogenesis is significantly regulated by VEGF, administration of anti-VEGF agents and VEGF inhibitors may offer a rational therapeutic approach in prostate cancer [20]. The primary finding in our study was the consequences of VEGF neutralization on tube formation and angiogenesis in HUVECs, our data showed that sFLT01 protein had robust anti-VEGF efficacy and inhibited capillary structures in in vitro angiogenesis examination. So, sFLT01 may play a key role in angiogenesis inhibition via VEGF neutralizing in endothelial cells. Proliferation dysregulation is one of the main characteristics of tumorigenesis. Data represented a strong correlation between the expression of sFLT01 and inhibition of DU145 cell proliferation. Other studies had previously shown that, sFLT01 was able to neutralize VEGF and block VEGF-stimulated HUVECs proliferation [17, 21] as well. HB-002.1 is a novel recombinant VEGF blocker with a significant dose-dependent inhibition on HUVECs proliferation and VEGF-induced tube formation when compared to that of bevacizumab function [22]. Another study reported that Bevacizumab inhibits the proliferation of A2780cis cells in ovarian cancer [23]. The AKT/ PI3K pathway and PTEN/PI3K signaling pathways regulate expression of VEGF and HIF-1α in prostate cancer cell lines. These pathways may contribute to tumor angiogenesis in this cancer [24, 25]. MEK/ERK/ RAF activation pathway is associated with endothelial cell proliferation may also contribute to progression of angiogenesis and invasiveness in solid tumors [26].

The fundamental process that contributes to tumor cell invasion, metastasis and also one of the critical steps of angiogenesis is endothelial cell migration [27]. We showed that, inhibition of VEGF by sFLT01 lead to reduced migration and decreased invasiveness of metastatic prostate cancer cells. A previously reported study revealed that, Luteolin blocked VEGF induced endothelial cells migration and invasion of prostate cancer in a dose-dependent manner [28]. Bevacizumab along with other anti-VEGF therapy impairs tumor invasiveness in a xenograft model due to increased SFK signaling pathway [29]. In combination with Atezolizumab, it dramatically reduces migration and invasion of A2780cis ovarian cancer cell line as well [23]. Apatinib is a tyrosine kinase inhibitor that effectively blocks VEGFR2. It suppresses migration and invasion in QBC939 and TFK-1 cells in cholangiocarcinoma (CCA) through modulatory effects on AKT and ERK/MEK/RAF signaling pathways [30]. VEGF activates downstream pathways including VEGFR-2/PI3K/Akt-PKB axis in endothelial cells, it follows activation of the small GTPases of the Rho family, PI3K and eNOS; SAPK2/p38; and phosphorylation of VEGFR-2 and FAK and then, endothelial cell migration occurs [31].

VEGF is one of the main inducers of GRP78 expression, which increases the proliferation ability of the vein endothelial cells [32]. Previous studies indicated that the growth, angiogenesis, and metastasis of cancer cells could be interrupted due to VEGF/GRP78 axis. GRP78 heterozygosity in transgenic-induced mice model of mammary tumors provokes apoptosis via increasing the CCAAT-enhancer-binding protein homologous protein (CHOP) expression and strong activation of procaspase-7 [33]. Accordingly, the growth of B16 melanoma metastatic lesions in mice carrying GRP78^+/−^ was significantly slower than wild-type GRP78 hosts [34]. Meanwhile, GRP78 silencing in endothelial cells significantly decreased tumor angiogenesis and proliferation in immortalized endothelial cells without any negative impact on healthy cells population [34]. Analysis of pancreatic ductal adenocarcinoma (PDAC) patients indicated significant expression of GRP78. Subsequent knocking out of GRP78 in PDAC cell lines affected tumoral proliferation and invasiveness by modulation of CyclinD1, CDK4, and CDK6 along with RhoA, ROCK1, JAK2, vimentin, Smad4, and p-STAT3 proteins [35]. The oncoprotein STAT3 is shown to provoke the PDAC invasion via upregulation of the MMP enzyme family expression and activity [36, 37].

Recent studies demonstrated that low glucose, hypoxia, and acidosis conditions in tumoral microenvironment could activate unfolded protein reaction and trigger GRP78 expression [38, 39]. In a hypoxic microenvironment, it has been suggested that GRP78 may be a downstream target of the HIF-1a gene. Anti-angiogenic molecules by reducing cell proliferation, hypoxia, and its involved factor (HIF-1a), lead to a decrease in the expression of GRP78 protein [40]. XBP-1-HIF-1a complex represents a crucial role in tumor growth and relapse by regulating genes involved in angiogenesis and metabolism such as VEGF, glucose transporter 1 (GLUT1), or pyruvate dehydrogenase kinase 1 (PDK1). Hypoxia leads to activation of angiogenic signaling pathways through production of the best-characterized pro-angiogenic factor VEGFA165 (VEGF). It has been shown that all three transcription factors of the UPR signaling pathway (spliced XBP-1, ATF4, and cleaved ATF6) have binding sites on the VEGF promoter region and enhance its transcription [38, 41, 42]. Considering reported experimental evidences in strong association between GRP78 and hypoxia as well as the anti-angiogenic properties of the sFLT01 molecule, it can be concluded that the sFLT01 molecule could inhibit the ER stress in a disease condition as like as tumor microenvironment which is suffering from severe hypoxia.

According to our data, sFLT01 protein showed modulatory impact on proliferation, invasion, and migration of DU145 cells along with the potential of HUVECs angiogenesis. Real-Time PCR analysis depicted a significant downregulation in GRP78, MMP2 and MMP9 transcripts’ levels, and a subsequent elevation of TIMP1 and TIMP2 expression under sFLT01 stimulation was detected. Moreover, there are some reports on the inhibitory effects of anti-VEGF drugs on ER stress indicators at hypoxic conditions in retinal pigmented epithelium cells (RPE) as well. Bevacizumab mitigated ER stress in human RPE cells cultured under hypoxic conditions. It reduced expression of two ER stress indicators, GRP78 and CHOP, under hypoxic conditions [43].

## Conclusion

As prescribed studies highlighted the critical role of GRP78 in PC development, the current investigation was made to study whether the anticancer impact of sFLT01 chimeric receptor could be mediated through suppressing the VEGF/GRP78 axis.

Our data showed that sFLT01 treatment also had a positive impact on TIMP1&2 expression. TIMPs as the known negative regulators of MMPs highly implicated in cancer malignancies [44, 45]. At the protein level, TIMPs could be targeted by multiple inhibitors, including GRP78, which has been shown to bind to TIMPs directly and forms GRP78-TIMP complex [45]. Accordingly, GRP78 inhibition with the Indolylkojyl methane analog IKM5 was recently reported to prevent GRP78-TIMP complex formation and abrogated invasiveness in breast cancer cells [46]. On the other hand, the expression level of TIMP1 in cancer cells treated with IKM5 was significantly higher than the control group, which is in line with our observations. Although the exact underlined mechanism remains unknown, one might speculate that TIMPs upregulation in cancer cells treated with GRP78 inhibitors might be mediated through transforming growth factor-β (TGF-β) stimulation, as recently reported by Cultrara et al. [11].

In summary, this study highlighted some anticancer aspects of sFLT01 as a next-generation antiangiogenic agent and showed that the inhibitory impact of sFLT01 on angiogenesis, growth, invasiveness, and migration of cancer cells could be mediated through the modulation of VEGF/GRP78/MMP2&9 axis and activation of TIMPs. For the first time, we demonstrated that sFLT01 protein is a novel therapeutic opportunity to suppress prostate tumor cells invasiveness.

However, more investigations are needed to elucidate the mechanism underlining sFLT01 anticancer activity.

## Materials and methods

### Cell culture

Human PC cell line DU145 along with the human embryonic kidneys (HEK293T) which were purchased from the National Institute of Genetic Engineering and Biotechnology cell bank (NIGEB Tehran, Iran) were considered for in vitro examinations. We used Roswell Park Memorial Institute (RPMI1640, Invitrogen, USA) and Dulbecco’s Modified Eagle Medium (DMEM, Gibco, USA) for culturing DU145 and HEK293T cells, respectively. Each media was supplemented with 100,000 U/L of penicillin, 100 μg/ml streptomycin (Fluka, Switzerland), and 10% fetal bovine serum (FBS, Biowest, France). Cells incubation was carried out in a humidified 5% CO2 incubator at 37 °C (Binder, USA).

### Vector construct and transfection

Human sFLT01 coding Sequence [21] was synthesized and His Tag sequence was added to the end of the construct with PCR reaction (primer sequences: 5′- GAATTCATGGTCAGCTACTG − 3′ (Forward), and 5′- GGATCCTCAGTGGTGGTGGTGGTGGTGTTTACCCGGAGACAGGGAG − 3′ (Reverse)). PCR product was cloned into pJET1.2/blunt plasmid (Thermo Fisher Scientific, Canada). The thermocycler was programmed as follows; 95 °C for 5 min denaturation step, and 35 cycles consisted of 95 °C for 30 s, 58 °C for 30 s, and 72 °C for 2 min following an additional 72 °C for 5 min, final extension step with Pfu DNA Polymerase (Agilent, USA).

PCR product was inserted into a pJET1.2/blunt plasmid (Thermo Fisher Scientific, Canada) and cloned in *Escherichia coli* XL10 bacteria (Agilent, USA) using the heat-shocked method [11]. After bacterial proliferation, the pJET-sFLT01-HisTag plasmids were extracted with the Plasmid Extraction Kit (SinaClon Co, Iran) according to the company protocol. After recovering the sFLT01-HisTag fragment from the gel, the fragment of interest was inserted into the pAAV-MCS-GFP vector (Agilent, USA) through a ligation protocol, and then the resulted plasmids were transformed into the host bacterial cells. Eventually, pAAV-sFLT01-HisTag-GFP plasmids were purified then analyzed with the gel electrophoresis method and sequences were determined.

Human PC cell line DU145 was considered as the host for transfection of pAAV-sFLT01-HisTag-GFP and pAAV-MCS-GFP vectors. 4 × 10^6^ cells seeded into each well of the six-well plates. Transfection was mediated by using the Lipofectamine 2000 reagent (Invitrogen, USA) according to the manufacturer’s guidelines. In brief, 5 μg of DNA sample was mixed with 525 μl of the antibiotic-free culture medium and added to a mixture of 21 μl lipofectamine 2000 reagent in 502 μl of culture medium. Following 20 min incubation at room temperature, the transfection mixture was gently dropped on the cells, admixed, and kept for 6 h at 37 °C under 5% CO_2_. After replacing the culturing medium, cells were incubated for the next 72 h.

### RNA isolation and real-time PCR analysis

Total RNA was isolated from DU145 treated and control cultures (DU145 cells transfected by pAAV-sFLT01-HisTag-GFP or control vector, pAAV-MCS-GFP) using the TriPure Isolation Reagent (Roche, Germany), according to the manufacturer’s protocol. The RNA samples were subsequently treated with 1 μg DNase I enzyme (Accurate genomic DNA removal kit, ABMgood, Canada) for 1 h and quantified with an ND-1000 Nanodrop (Nanodrop Technologies, USA).

1 μg of total RNA was applied for cDNA synthesis by the QuantiTect Reverse Transcription Kit (Qiagen Inc., USA). Real time-PCR was carried out on a 7500 Real-Time PCR System (Applied Biosystems, USA) with the QuantiFast SYBR Green PCR Kit (Qiagen Inc., USA). The thermocycler was programmed as follows; a 95 °C for 3 min denaturation step, and 40 cycles consisted of 95 °C for 10 s and 60 °C for 30 s. The expression level of sFLT01 was normalized to GAPDH as an endogenous control and the expression level of interested genes was calculated by using the 2^-∆∆CT^ method based on the threshold cycle (Ct) values. Each sample was assessed in duplicate at least. The primer sequences were as follows: sFLT01 Forward 5′-AGGAAGGGAGCTCGTCATTC-3′ and Reverse 5′-GCCCATTGACTGTTGCTTCA-3′, GRP78 Forward 5′- CGTGGAGATCATCGCCAAC-3′ and Reverse 5′-ACATAGGACGGCGTGATGC-3′, MMP2 Forward 5′-TTGATGGCATCGCTCAGATC-3′ and Reverse 5′-TTGTCACGTGGCGTCACAGT-3′, MMP9 Forward 5′- GTGATTGACGACGCCTTT − 3′ and Reverse 5′- CAACTCGTCATCGTCG-3′, TIMP1 Forward 5′- CTTCTGGCATCCTGTTGT-3′ and Reverse 5′- ACTGCAGGTAGTGATGTG-3′, TIMP2 Forward 5′-AAGCGGTCAGTGAGAAGGAAG-3′ and Reverse 5′- GGGGCCGTGTAGATAAACTCTAT-3′, and GAPDH Forward 5′-ACAGTCAGCCGCATCTTC-3′ and Reverse 5′- CTCCGACCTTCACCTTCC-3′.

### Purification and evaluation of sFLT01 protein

To determine the sFLT01 protein concentration in transfected HEK293T cells. Culture medium was collected 72 h after transfection and centrifuged for 10 min at 500×g to remove the bulk of cell debris. His-tagged sFLT01 protein was purified using the nickel affinity chromatography (Ni–NTA agarose beads, ABT, Spain), according to the company protocols. Next, SDS-PAGE and Western blotting techniques were applied to determine the level of sFLT01 protein using 1:50000 human VEGFR1/Flt-1 primary antibody and a 1:100000 goat IgG HRP-conjugated secondary antibody (R&D Systems, USA). Blots were developed with using ECL select™ Western blotting detection reagent (GE Healthcare, Amersham™,Buckinghamshire HP7 9NA UK) and a specific band was visualized.

### Cellular viability assay (MTT)

Viability of cell cultures was determined by the MTT colorimetric assay (Sigma-Aldrich, USA). A total of 6.6 × 10^3^ DU145 cells/well were seeded into each well of a 96-well plate and incubated with 100 μl media containing RPMI1640 and DU145 cells conditioned medium in a ratio of 2:1 (DU145 cells transfected by pAAV-sFLT01-HisTag-GFP or control pAAV-MCS-GFP transfected DU145 cells), for 48 h at 37 °C under 5% CO_2_. Then, each well-received 20 μl of MTT (0.5 mg/ml in PBS, pH 7.2) and cultures were kept for the next 4 h. After removing the solution, cells were treated with 200 μl of dimethyl sulfoxide (DMSO, Sigma-Aldrich, USA) for 5 min. The number of viable cells was assessed using an ELx800 absorbance microplate reader (Bio-Rad, USA), at a wavelength of 580 nm.

### Tube formation assay

Tube formation assay was performed to determine the ability of sFLT01 to inhibit tube formation in the human umbilical vein endothelial cells (HUVECs). The 96-well plates were coated with growth factor-reduced Matrigel (BD Bioscience, Belgium), then HUVECs (3.5 × 10^4^ cells/well) seeded on top of Matrigel-coated wells. After 24 h, collected CMs from DU145 cells transfected by pAAV-sFLT01-HisTag-GFP or control, pAAV-MCS-GFP containing construct (supplemented with 10% FBS) were added and incubated for 18 h. To compare tube formation ability of groups, the number of master junctions along with the number of meshes from four randomly picked spots were evaluated under an inverted phase-contrast microscope (Olympus, Japan) and quantified with ImageJ software (AngioTool plugin).

### Wound healing assay and cell invasion assay

To investigate the impact of sFLT01 on cancer cells migration, DU145 cells were seeded in a six-well plate at a density of 2 × 10^5^ per well, and cultured for 24 h. When cultures developed to a near monolayer, a wound was made using the tip of a pipette and cells were treated with CM (from treated DU145 transfected by pAAV-sFLT01-HisTag-GFP or control which transected by pAAV-MCS-GFP). Following 24–48 h incubation, cells were fixed in a 3% formaldehyde solution for 15 min and imaged using different fields. The relative migration rates of DU145 cells were determined by dividing the migration distance by the time.

Invasion analysis was performed by using matrigel (BD Biosciences, San Jose, CA, USA) was added to the underside of each insert and left for 16 h at 37 °C in a 5% CO 2 atm. According to the manufacturer’s instruction. First, a mixture of 5 × 10^5^ DU145 cells transfected by pAAV-sFLT01-HisTag-GFP or, control, pAAV-MCS-GFP transfected DU145 cells and serum-free media was prepared and added to the matrigel-coated transwell chambers (Nunc, Roskilde, Denmark) with 8.0 μm diameter pores. The chambers were then placed in the lower plate filled with 10% FBS containing media and maintained for 24 h at 37 °C under 5% CO_2_. After removing the non-invading cells, the cells were fixed in the chilled methanol for 20 min at − 20 °C. The plate was rewashed with the PBS buffer twice and stained with DAPI for the next 3 min. Cell analysis was carried out using a fluorescent microscope (Carl Zeiss, Germany).

## Data Availability

The data that support the findings of this study are available on request from the corresponding author. The data are not publicly available due to privacy or ethical restrictions.
